# Empirical comparison of genotoxic potency estimations: the *in vitro* DNA-damage ToxTracker endpoints versus the *in vivo* micronucleus assay

**DOI:** 10.1093/mutage/geab020

**Published:** 2021-06-10

**Authors:** John W Wills, Elias Halkes-Wellstead, Huw D Summers, Paul Rees, George E Johnson

**Affiliations:** 1 Biominerals Research Group, Department of Veterinary Medicine, Cambridge University, Cambridge, UK; 2 Centre for Nanohealth, Swansea University College of Engineering, Swansea, UK; 3 Institute of Life Science, Swansea University Medical School, Swansea, UK; 4 Broad Institute of MIT and Harvard, Cambridge, MA 02142, USA

## Abstract

Genetic toxicology is an essential component of compound safety assessment. In the face of a barrage of new compounds, higher throughput, less ethically divisive *in vitro* approaches capable of effective, human-relevant hazard identification and prioritisation are increasingly important. One such approach is the ToxTracker assay, which utilises murine stem cell lines equipped with green fluorescent protein (GFP)-reporter gene constructs that each inform on distinct aspects of cellular perturbation. Encouragingly, ToxTracker has shown improved sensitivity and specificity for the detection of known *in vivo* genotoxicants when compared to existing ‘standard battery’ *in vitro* tests. At the current time however, quantitative genotoxic potency correlations between ToxTracker and well-recognised *in vivo* tests are not yet available. Here we use dose–response data from the three DNA-damage-focused ToxTracker endpoints and from the *in vivo* micronucleus assay to carry out quantitative, genotoxic potency estimations for a range of aromatic amine and alkylating agents using the benchmark dose (BMD) approach. This strategy, using both the exponential and the Hill BMD model families, was found to produce robust, visually intuitive and similarly ordered genotoxic potency rankings for 17 compounds across the BSCL2-GFP, RTKN-GFP and BTG2-GFP ToxTracker endpoints. Eleven compounds were similarly assessed using data from the *in vivo* micronucleus assay. Cross-systems genotoxic potency correlations for the eight matched compounds demonstrated *in vitro*–*in vivo* correlation, albeit with marked scatter across compounds. No evidence for distinct differences in the sensitivity of the three ToxTracker endpoints was found. The presented analyses show that quantitative potency determinations from *in vitro* data enable more than just qualitative screening and hazard identification in genetic toxicology.

## Introduction

Genetic toxicology is an essential component of compound safety assessment, with the aim of ensuring that the risk of adverse human health effects caused by DNA damage is minimised. Traditionally, genetic toxicity testing has only been used for hazard identification and the screening of compounds into simple ‘positive’ or ‘negative’ groups. However, the limitations of this approach, alongside the realisation, that much more information can be gained from genetic toxicity dose–response data are increasingly recognised. The alternative, quantitative paradigm uses dose–response analysis of genetic toxicity endpoints to determine point-of-departure values below which the risks posed by the small increase in adverse effects can be considered negligible (1–6).

To achieve this, multiple working groups and research outputs have evaluated different quantitative methodologies for the assessment of genetic toxicity dose–response data (7,8). A major conclusion of this work has been that the ‘benchmark dose’ (BMD) approach is both well-suited and easily accessible through the provision of open-source software. The BMD approach operates by nonlinear regression analysis—fitting a function to dose–response datasets under consideration and then interpolating to define the ‘benchmark dose’ (i.e. the equipotent dose) that can be expected to cause a predefined increase relative to the negative control (e.g. 50%). Importantly, by considering the range of fits compatible with the dose–response data, the uncertainty in the estimation of the BMD can be established, allowing expression in terms of lower (BMDL) and upper (BMDU) two-sided 90% BMD confidence interval (2,9,10).

**Fig. 1.**
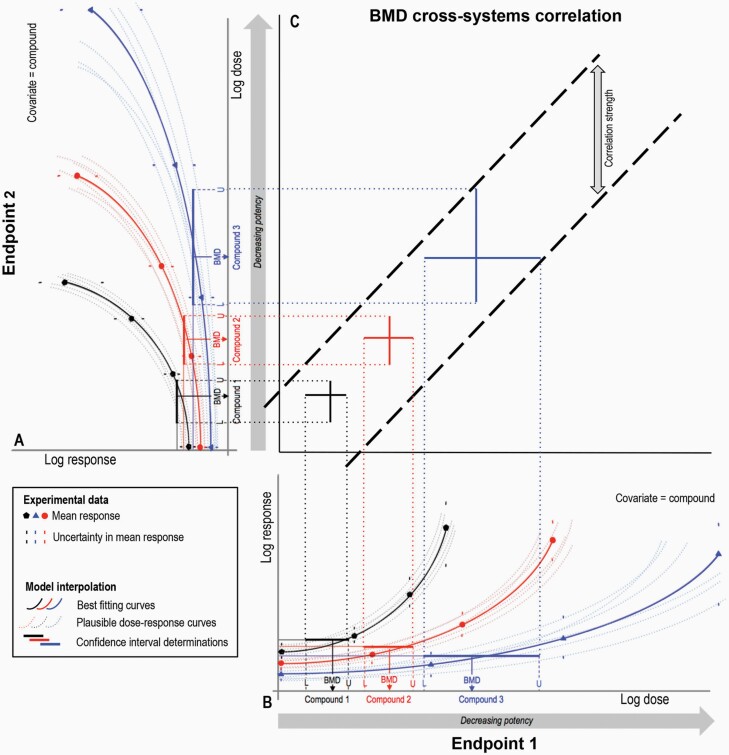
Schematic overview explaining BMD-derived, cross-system genotoxic potency correlation. (**A and B)** For the dose–response data available for each end point (e.g. arising from multiple compounds), the BMD approach provides an estimate of the ‘equipotent dose’ that can be expected to elicit a predetermined effect size (termed the BMR) relative to control response. In addition to finding the best-fitting curve to describe each dose–response relationship (solid curves), other fits that could also plausibly describe the data are shown (dashed curves), and together these allow calculation of the 90% two-sided BMD confidence interval (i.e. BMDL (L)–BMDU (U), represented schematically here by the solid coloured lines). In this way, the confidence interval represents the uncertainty in estimation of each BMD upon the basis of the available dose–response data. (**C)** For matching compounds across endpoints, these BMD confidence intervals can then be plotted against one another on the X and Y axes (e.g. across *in vitro* and *in vivo* systems) to check for correlation. If the correlated intervals scatter randomly between two bounding diagonals drawn with unity slope (i.e. slope equal to 1), this is evidence of a proportional correlation between the two systems under comparison on original scales (see Materials and methods for further explanation). In turn, the vertical distance between the bounding diagonals represents a basic measure of the strength of the correlation.

More recently, computational approaches (e.g. the PROAST software) made freely available by researchers at the Dutch National Institute of Public Health and the Environment (RIVM) allow combined analysis of multiple dose–response datasets across covariate subgroupings included in the analysis (e.g. compound, sex, strain, exposure regimen, cell type, genotype etc.) (1,2,9). Importantly, these combined analyses have the potential to yield more precise BMD estimates in instances where one or more of the fitted model parameters can be considered the same at the covariate level, enabling estimation of these conserved parameters from the combined dose–response datasets (9). As a result of the open availability of these techniques, a growing body of work has shown that BMD estimates and their confidence intervals are extremely well-suited for enabling robust potency comparisons within endpoints—as well as enabling empirical potency comparisons across endpoints (schematically explained in Figure 1) (1–4,6,11).

An important application for cross-end point comparison is the ability to compare the results obtained from higher throughput *in vitro* methods with data collected from animal models. This work is considered extremely important for improving understanding and expanding the application domain of *in vitro* methods (2,6). This is because, at least for the time being, *in vitro* approaches are considered best suited for hazard identification and prioritisation purposes, in addition to aiding compound mode-of-action determinations (2,6). Moving *in vitro* data beyond this into the domain of human health risk assessment is generally considered extremely challenging for a multitude of reasons including the complexities of tissue-specific metabolism and toxicokinetics (1,2,6). Nonetheless, it is established that empirical BMD comparisons between *in vitro* and *in vivo* systems can establish *if* correlations exist, in addition to variability that can be expected across compounds and across different endpoints (6,11–13). In this way, such work directly informs on the utility of *in vitro* data for *in vivo* extrapolation purposes and, perhaps more importantly, can demonstrate the utility of simpler, less ethically divisive *in vitro* approaches for providing effective compound potency rankings that contribute to human-relevant risk assessments (2,6).

Historically, *in vitro* genotoxicity testing has relied heavily on the Ames bacterial mutation test followed by a mutation test and chromosome damage assay in mammalian cells. This ‘standard battery’ approach is known to exhibit relatively low specificity, placing importance on the development of new approaches to improve safety assessments in the face of the ever-increasing barrage of new compounds (5,14,15). One such approach is the ToxTracker assay (16,17), which uses six genetically stable, mouse embryonic stem cell lines each with a green fluorescent protein (GFP) gene construct reporting on a distinct aspect of cellular perturbation. The six cell lines can be loosely placed into four categories on the basis of mechanism correlated with gene pathway—namely DNA damage, p53-mediated cellular stress, oxidative stress and protein stress (16,17). Importantly, the ToxTracker assay has already been shown to provide improved sensitivity and specificity for the detection of known *in vivo* genotoxins and rodent carcinogens when compared with other *in vitro* alternatives, including the Ames, *in vitro* micronucleus and chromosomal aberration tests (16,18). At the current time however, empirical comparisons against dose–response relationships quantitatively assessed from well-recognised *in vivo* tests are not yet available.

To this end, here we focus on the three ToxTracker endpoints that specifically report on DNA-damage-inducible pathways (including global p53 up-regulation). These are BSCL2-GFP, RTKN-GFP and BTG2-GFP cell lines described extensively in previously published work (16,17). In brief, BSCL2-GFP is associated with the ataxia telangiectasia and Rad3-related and checkpoint kinase 1 DNA damage signalling pathway well-known to modulate DNA replication, influence cell cycle stalling and induce apoptotic cell death. RTKN-GFP is associated with the NF-κB signalling pathway and is up-regulated in response to the formation of DNA double-strand breaks following exposure to wide-ranging DNA damaging agents. Finally, BTG2-GFP expression reflects activation of the global p53 response—and can therefore be induced by both DNA damage and oxidative stress (16,17). Using dose–response data from these reporters as well as from the *in vivo* micronucleus assay, we carry out BMD analyses for a range of aromatic amine and alkylating agents—many of specific relevance as pharmaceutical products or known genotoxic impurities. We investigate the utility of the BMD approach to provide robust compound potency rankings within each end point and then use empirical comparison across the *in vitro* and *in vivo* systems to establish (i) whether correlations exist and (ii) the relative sensitivity of each of the ToxTracker endpoints under study.

## Materials and methods

### Compound abbreviations

2-acetylaminofluorene = AAF; 4-aminobiphenyl = ABP; 5-azacytidine = ACD; allyl bromide = ALB; benzo[*a*]pyrene = BAP; chlorambucil = CHAMB; 2-chloroethanol = CHO; cisplatin = CIS; cyclophosphamide = CPA; ethyl methanesulphonate = EMS; N-nitroso-N-ethylurea = ENU; hydroquinone = HYD; mitomycin C = MMC; methyl methanesulphonate = MMS; N-nitrosodimethylamine = NDMA; 2-amino-1-methyl-6-phenylimidazo[4,5-b]pyridine = PHIP; zidovudine = ZVD.

### Data sources

Flow cytometry-based dose–response data for the ToxTracker endpoints were provided by Toxys. The supporting ToxTracker methodology is extensively described in previous publications (16,17). *In vivo* micronucleus frequency data were collected from previously published studies—many of which were available due to the efforts of the Health and Environmental Sciences Institute Quantitative Analysis Workgroup (QAW) to compile dose–response information into an accessible format. Dose–response data for AAF were collected from Asano *et al.* (19). ABP was taken from Shelby *et al.* (20). ACD was collected from the US National Toxicology Programme (NTP) study number A95392. Information for ALB was combined from NTP studies A43640, A87628 and A03068. Data for BAP were collected from Shimada *et al.* (21). The data used for CHAMB are described in Dertinger *et al.* (22). Data for CHO were collected from NTP study number 666681. CPA dose–response data were collected from the studies described in Goralick *et al.* (23), Vrzoc *et al.* (24) and Hatanaka *et al.* (25). Data for EMS and ENU were collected from Gocke *et al.* (26). Data for MMS were collected from Ji *et al.* (27).

### BMD analyses

For the ToxTracker endpoints, after visually inspecting the dose–response curves for erratic response values that occurred concomitantly with high cytotoxicity values, a cell survival cut-off was imposed on the data taken forward for BMD modelling at 40%. The PROAST (version 65.5) (http://www.proast.nl) R-package was used to carry out the BMD analyses. As default, PROAST performs analyses on log_10_ transformed data because previous analyses of wide-ranging dose–response data across diverse toxicological endpoints have consistently demonstrated multiplicativity as opposed to additivity (9). This is to say that responses occur relative to the current value, not by the same absolute amount. For this reason, the BMD results are displayed on log_10_ scales, such that the same percent change is visually the same at any position on the axis (9). To test this assumption of log-normality with the ToxTracker dose–response data, quantile–quantile (qq) plots of model fit residuals against theoretical quantiles were performed within the PROAST package during data preprocessing (presented in Supplementary Figure S1, available at *Mutagenesis* Online). Consistent with the presented qq plots, any minor deviations from log-normality could reasonably be expected to have minimal impact on the ‘coverage’ of the calculated BMD confidence intervals. In this regard, it is also recognised that robust assessments of the distribution of biological data are always complicated by ubiquity of non-random errors unavoidably present since it is not practically feasible to randomise all experimental conditions and concomitant treatments (9).

Dose–response data were analysed using either the exponential or the exponential and Hill model families that are recommended for the assessment of continuous toxicity data by the European Food Safety Authority (EFSA) (10). In each analysis, combined datasets (i.e. across compounds) were analysed together using the ‘combined covariate’ approach, with compound specified as a potential covariate. Models with additional parameters were accepted if the fit significantly (*P* < 0.05; log-likelihood) improved. This process allowed the model parameters that could be considered constant across subgroups and those which needed estimating for each subgroup to be established. Here, in keeping with previous findings (9), it was found that the log-steepness (*parameter d*) and maximum response (*parameter c*) could reasonably be held equal for all response curves, whereas the parameters for potency (*parameter b*), background response (*parameter a*) and within-group variance (*var*) were examined for covariate dependency. PROAST outputs describe potency in terms of the ‘benchmark dose’ (i.e. the equipotent dose) in addition to describing the two-sided 90% confidence interval (i.e. the BMDL and BMDU, respectively) for each level of the covariate. Fitted models to all dose–response data are shown in Supplementary Figures S2–S5, available at *Mutagenesis* Online. The benchmark response (BMR) size used in all analyses was 50%, which equates to a 50% increase in response relative to the background established in the negative, (zero-dose) control. The BMDL and BMDU represent the lower and upper limits of the two-sided 90% confidence interval of the BMD, with the ‘quantity’ of the confidence interval therefore representing the precision of its estimation. Plots of confidence intervals organised by midpoint were used within biomarker endpoints to present a visually intuitive potency comparison across compounds that is also representative of the uncertainties present in the underlying dose–response relationships (1,2).

### Cross-system correlation

To correlate matching compounds across *in vitro* and *in vivo* systems, the BMD-derived confidence intervals per system were plotted against one another. As established in previous work (6,11–13), two boundary lines encompassing all compounds were then drawn with a slope of 1 (i.e. unity slope) on the double-log scale. Only if the compounds scatter evenly between these boundary lines is there evidence of a proportional relationship between the systems on the original scale: This is because if, on original scale, *y* = *bx* ^ *c* (where ‘^’ indicates the exponent operator), then on the double-log scale, we have log(*y*) *=* log(*b*) + *c* log(*x*). Importantly, this shows that *c*, the slope in the double-log plot, is the power of *c* on original scales. For this reason, when the correlation shows agreement with *c* = 1 on the log-scale, there is evidence of a proportional relationship between the *in vitro* and *in vivo* systems under comparison. In this way, the vertical distance between the boundary diagonal lines represents a basic measure of the strength of the correlation (6). In turn, where support for a proportional correlation is found, the Y-axis intercept and the error in its estimation provides a measure of the sensitivity of the X-axis end point relative to the Y-axis end point. That is, under unity slope, an increasing Y-axis intercept for the correlation indicates potential greater sensitivity in the end point represented on the X-axis.

### Linear regression accounting for measurement error in X and Y directions

To estimate the Y-axis intercept for the correlated endpoints, we use an algorithm developed by York *et al.* (28,29) to perform linear regression on the data that importantly accounts for the error in X and Y directions at each point. The method assumes a normal distribution of the data about each point in both X and Y directions.

## Results and discussion

Previous work has demonstrated the importance of comparing BMD confidence intervals (i.e. the interval between the BMDL and BMDU) instead of just comparing BMD values when assessing BMD results, such as genotoxic potency estimations arising from different compounds (1,2,4). Considering the interval, as opposed to just the ‘point value’ BMD estimate, is important because it defines the range within which we are most assured that the true BMD lies. In this way, the interval reflects the *uncertainty* with which we are able to estimate the BMD on the basis of the precision and ‘quality’ of the underlying dose–response relationship (Figure 1). A further consequence of this is that BMD values across compounds should only be considered significantly different when confidence intervals do not overlap. When overlaps do occur, the interpretation is that the underlying dose–response relationships do not contain sufficient information to define how potencies differ. In turn, when a set of overlapping confidence intervals occupy a sufficiently narrow range, the potency estimations may be considered similar enough to evidence equipotency (1,2,4).

**Fig. 2.**
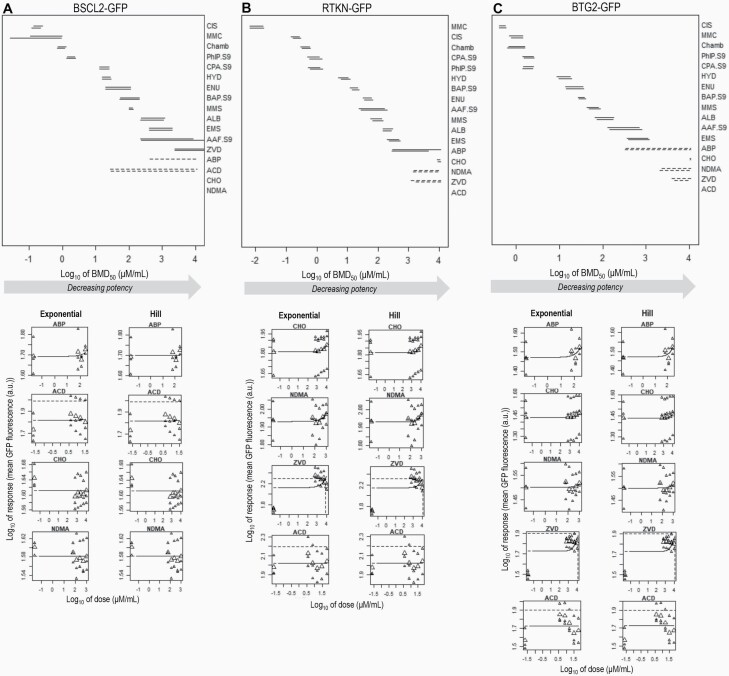
BMD-derived potency rankings for 17 compounds using dose–response data from three *in vitro* ToxTracker reporter cell lines. (**A–C**) The upper panels show the two-sided 90% confidence intervals (CIs) for the BMD_50_ defined for each compound using dose–response data from the (A) BSCL2-GFP, (B) RTKN-GFP or (C) BTG2-GFP ToxTracker reporters. The suffix ‘.S9’ indicates compounds tested using supplemental metabolic activation. For each compound, the upper and lower intervals reflect the BMD results obtained using the exponential or the Hill model families, respectively. Dashed CIs represent BMDs where the upper confidence limit of the BMD (i.e. the BMDU) could not be determined from the available dose–response data. Missing CIs reflect BMDs for which the dose–response data did not permit estimation of either the BMDL or the BMDU (i.e. infinite lower and upper CIs). In reading the potency ranking, overlapping CIs between compounds reflect potencies that cannot be resolved due to uncertainties in the available dose–response data. The lower panel shows the underlying dose–response data and fitted model (solid line) for BMDs with unbounded CIs. Horizontal and vertical dashed lines represent interpolation at the critical effect size (i.e. BMR_50%_) to define the BMD_50_ (respectively). All underlying dose–response data and fitted model curves are shown in Supplementary Figures S2 and S3, available at *Mutagenesis* Online.

In Figure 2, this concept is applied to derive genotoxic potency estimations for 17 alkylating agent/aromatic amine compounds—many with known genotoxic activities—using *in vitro* dose–response data from the three DNA-damage-focused ToxTracker reporter endpoints (BSCL2-GFP, RTKN-GFP and BTG2-GFP). For each compound, two confidence intervals are shown, one derived using the exponential model family, the other using the Hill model family (BMD analyses and model fits shown in Supplementary Figure S2 and S3, available at *Mutagenesis* Online). In many instances, the ToxTracker dose–response data determined by high-throughput flow cytometry measurements of GFP expression per-cell for 5+ dose groups are seen to enable precise estimation of the BMD, with many of the confidence intervals spanning <0.5 log units (3-fold ratio on original scales). It is also striking that despite each reporter feeding back on distinct aspects of the DNA damage response pathway, the compound potency rankings across reporters followed similar orders (Figure 2A–C). To demonstrate that this was not a result of the combined BMD modelling approach used, the dose–response relationships for all compounds were also modelled independently (i.e. one at a time in series) (presented, Supplementary Figure S6, available at *Mutagenesis* Online) (1,2). This approach still yielded highly similar compound potency rankings across the three endpoints—albeit with markedly wider confidence intervals (1,2). It therefore seems likely that the similarities across endpoint potency rankings speak to significant overlaps in pathway activation upon DNA damage induction (16). To test this a step further, matched data for the same set of compounds were also modelled for the DDIT3-GFP ToxTracker endpoint, which reports on protein stress as opposed to DNA damage. This time, as expected, assessment with the combined BMD modelling approach confirmed a very different potency ranking to that observed for the DNA damage endpoints (shown in Supplementary Figure S7, available at *Mutagenesis* Online).

Returning to the similarities observed across the DNA damage endpoints, an exception appears to be the particularly low BMD estimate for the highly reactive DNA cross-linker, MMC via the RTKN-GFP reporter. Here perhaps, the heightened sensitivity of this reporter to this compound makes sense, given that its feedback is closely linked to the initiation of DNA double-strand breaks (Figure 2B). Across all three reporters, a combination of the dose–response data for the compounds NDMA/ACD/CHO and/or ZVD/ABP yielded confidence intervals with either unbounded upper limits (i.e. incalculable BMDUs) or unbounded upper and lower limits (i.e. infinite confidence intervals as a result of incalculable BMDLs and BMDUs). Inspection of the underlying dose–response relationships (Figure 2, lower panels) showed in all instances that this arises due to weak/unclear responses established across the tested dose ranges. Nonetheless, considering the outputs across the three DNA damage ToxTracker endpoints, it is worth noting that at minimum, a BMDL was determinable for all five of these compounds by at least one of the endpoints. This matters because even when the BMDU cannot be defined, the BMDL tells us that *if* there is an effect, it will likely occur for doses above the BMDL (1,2,30,31). Similarly, while BMD estimations for ABP via the BSCL2-GFP reporter yielded infinite confidence intervals using the Hill model, a BMDL was established using the exponential model (Figure 2A). This shows the value of including both model families when carrying out BMD-derived potency estimations.

**Fig. 3.**
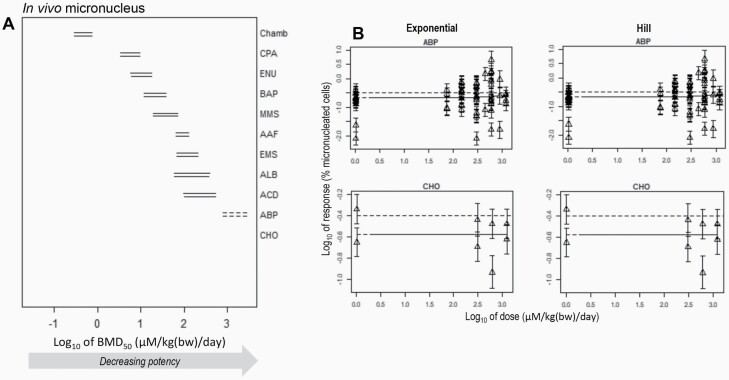
BMD-derived potency rankings for 11 compounds using dose–response data from the *in vivo* micronucleus assay. (**A**), The left panel shows the two-sided 90% confidence interval (CI) for the BMD_50_ defined for each compound. In turn, the upper and lower intervals reflect the BMD results obtained using either the exponential or the Hill model family, respectively. Dashed CIs represent BMDs where the upper confidence limit of the BMD (i.e. the BMDU) could not be determined. Missing CIs reflect BMDs for which the available dose–response data did not permit estimation of either the BMDL or the BMDU. (**B**) The right panel shows the underlying dose–response data and fitted model (solid line) for BMDs with unbounded CIs. The horizontal dashed lines represent interpolation at the critical effect size (i.e. BMR_50%_). The BMD analyses and all underlying dose–response data and fitted model curves are shown in Supplementary Figures S4 and S5, available at *Mutagenesis* Online.

In the same way, Figure 3A shows the results from BMD analysis of 11 similar aromatic amine and alkylating agent compounds, but this time using dose–response data from the *in vivo* micronucleus test. Bounded confidence intervals (i.e. BMDLs and BMDUs) were determinable for nine of the compounds (BMD analyses and model fits shown in Supplementary Figure S4 and S5, available at *Mutagenesis* Online). For ABP, a BMDL could be determined, but the BMDU was infinite, whereas for CHO both the BMDL and BMDU were incalculable. Again, consideration of the underlying dose–response data shows why this arose (Figure 3B). At best, some evidence of a weak response was observed in the data for ABP, whereas no discernable response relative to vehicle control was found for the dose range tested for CHO (Figure 3B).

**Fig. 4.**
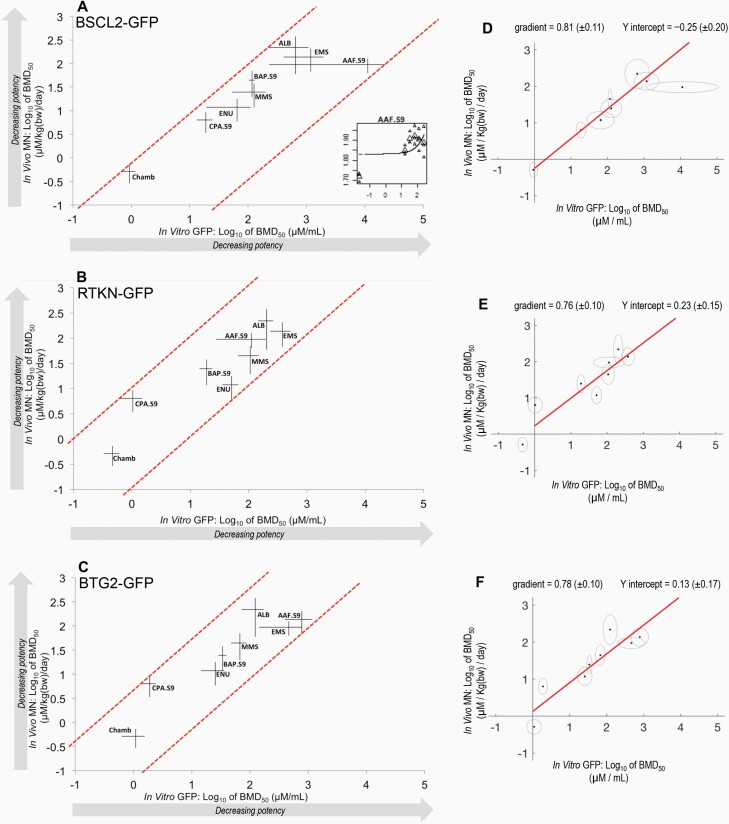
BMD-derived genotoxic potency correlations for three *in vitro* ToxTracker reporters versus the *in vivo* micronucleus assay for eight compounds. (**A–C**) Using the exponential model family, two-sided 90% confidence intervals (CIs) for the BMD_50_ for each chemical are shown as the horizontal (*in vitro*) and vertical (*in vivo*) lines, which connect at the BMD_50_ for each data point. (A) (inset) shows the uncertainty in the underlying *in vitro* dose–response data for compound ‘AAF’. For each comparison, the compound potency correlation is represented by the dashed diagonal lines which have unity slope and encompass all confidence intervals for the compounds under study. The suffix ‘.S9’ denotes compounds that were tested using supplemental metabolic activation *in vitro*. (**D–F**) York least-squares linear regression for the compound potency correlations shown in (A–C). In each plot, the red line represents the fit to the data when the uncertainty (grey ellipses) in both the X and Y direction is taken into account.

The analyses conducted in Figures 2 and 3 provide the opportunity to compare the *in vitro* genotoxic potency estimates for the three ToxTracker endpoints with the *in vivo* estimates from the well-established *in vivo* micronucleus test by cross-systems correlation (explained in Figure 1). To do this, BMD confidence intervals for the eight matched compounds where the underlying dose–response data allowed estimation of bounded BMDs were correlated against one another across the *in vitro* and *in vivo* systems (Figure 4).

To quantify the resulting correlation, diagonal lines were plotted such that they encompassed the BMD confidence intervals from both endpoints for all compounds (4,6,11–13,32). These bounding diagonals were drawn with a slope of 1 on the double-log_10_ plot. Importantly, agreement with this unity slope evidences a proportional relationship between the two correlated endpoints on original scales (4,6,11–13,32) (see Materials and methods). For all three ToxTracker endpoints, the genotoxic potency estimations across systems showed agreement with the slope of the bounding diagonals, providing evidence for a proportional, correlated relationship between the *in vitro* and *in vivo* systems—albeit with considerable scatter across compounds. To better understand this, the vertical distance between the two bounding diagonals was quantified, as this represents a basic measure of the strength of the correlation across test systems (6,12). For BSCL2-GFP (Figure 4A), BTG2-GFP (Figure 4B) and RTKN-GFP (Figure 4C), ToxTracker endpoints versus the *in vivo* micronucleus assay, these distances were 2.4, 1.9 and 2.0 log units, respectively (i.e. ~80- to 250-fold). When considering the apparent weaker correlation for BSCL2-GFP, it is important to note that this is largely driven by one compound, AAF, which exhibits a much wider confidence interval *in vitro* than is seen for any of the other compounds (the uncertainty in the underlying dose–response data that leads to this is shown in Figure 3A, inset). Similarly, but with more precisely defined potency estimations, CPA is observed to lie further away from the general trend in the BTG2-GFP and RTKN-GFP correlations suggesting heightened potency in these *in vitro* test systems compared to *in vivo* (Figure 4B and C).

With correlations in agreement with unity slope, the Y-intercept of the correlation on the double-log axes provides information on the relative sensitivity of each of the three *in vitro* endpoints under study. That is, under similar slope, the end point represented on the X-axis may be considered more sensitive as the Y-intercept increases. To assess this for the three *in vitro* ToxTracker endpoints under study here, the compound-matched correlations to the *in vivo* micronucleus data were fitted using the York least-squares estimation approach (Figure 4D–F). This returned a linear regression for each correlation that importantly takes into account the uncertainty represented in the BMD confidence intervals in both the X and Y directions. This fitting analysis again confirmed the similar slope—approaching unity—for each correlation. It also showed similar Y-axis intercepts suggesting similar sensitivities for the three ToxTracker endpoints under study. This was particularly true for RTKN-GFP and BTG2-GFP (Figure 4E and F), whereas the BSCL2-GFP intercept was slightly lower (~0.5 log units or 3-fold) (Figure 4D).

## Conclusions

Many of the compounds under study in this work are pharmaceutical products or impurities known to be able to cause DNA damage and thus contribute towards the initiation of cancer. Previously, the work of Hernandez *et al*. has shown—using extensive compound data and similar quantitative methodologies—a positive relationship between the results of multiple, shorter-term *in vivo* genotoxicity assays and the 2-year cancer bioassay (12,13). This pioneering work sets the precedent that comparatively simpler, shorter-term genotoxicity assays can be used to obtain meaningful information about carcinogenic potency (6).

Whereas this concept will in itself lead to the more effective use of animals to quantitate cancer risk, the growing global trend towards animal reduction necessitates the development of *in vitro* alternatives (2,33,34). For this purpose, the high-throughput, multi-end point ToxTracker assay appears promising for expediting genetic toxicity assessments, especially as validation studies (16) have shown it can achieve better sensitivity and specificity for the detection of known *in vivo* genotoxins than other *in vitro* alternatives. Importantly, here we show that dose–response relationships from the three DNA-damage focused ToxTracker reporters provide quantitative genotoxic potency estimates that correlate with those obtained from the *in vivo* micronucleus assay. This is to say—compounds that are potent micronucleus inducers *in vivo* are shown here to also be potent activators of the BSCL2-GFP, BTG2-GFP and RTKN-GFP reporter constructs used to biomonitor DNA damage responses in ToxTracker. As the *in vivo* micronucleus assay could be considered a ‘gold standard’ for the sensitive, human-relevant detection of chromosomal damage, the existence of these correlations further demonstrates the utility of the ToxTracker assay.

We now suggest that follow-up work with expanded numbers of compounds is extremely important. Access to more data may identify roles for toxicokinetics and compound-specific metabolism on the strength of the correlation, or could reveal compound-specific subgroupings within correlations or across reporters. Whereas such findings would be important to the continuing development and validation of ToxTracker, they are also of consequence for improving our understanding and utilisation of *in vitro* to *in vivo* extrapolation approaches more generally (6,16,17,32). This is because their existence shows that genotoxic potency information can be derived from both animal studies or cell culture models. Fully understanding this strengthens the use of *in vitro* systems as a routine component of regulatory review for preclinical safety submissions of pharmaceuticals and other consumer or industrial products. It may also allow *in vitro* data to contribute more directly to product safety investigations, regulatory decision-making and human health risk assessment (2,6).

More generally, the quantitative assessment of genetic toxicity data is a rapidly advancing field, and the use of the BMD approach to derive point-of-departure values offers significant advantages for regulatory decision-making and the protection of human health (7,8). The analyses presented in this work again show that *in vitro* data have far more utility than just qualitative screening and hazard identification in genetic toxicology (4).

## Supplementary data

Supplementary data are available at *Mutagenesis* Online.

Figure S1—Quantile-quantile (qq) plots for exponential or Hill model fits to the *in vitro* ToxTracker dose-response data for 17 compound. Each plot shows theoretical quantiles plotted against the observed quantiles after model fitting. Points represent residuals where significant deviations from the line indicate deviation from the theoretical log-normal distribution. *N.b.,* it should be noted that the ‘tails’ can be expected to deviate from the line even if the assumption holds perfectly.

Figure S2—BMD analyses using exponential or Hill model families for 17 compounds using dose-response data from the *in vitro* DNA-damage ToxTracker endpoints. Combined datasets were analysed using ‘compound’ as covariate. In each instance, the data were adequately described using a single exponential curve with constant parameters for max-response (*c*) and log-steepness (*d*). Note that the control group (*i.e.,* dose zero) is situated at minus infinity on a log10-scale and a ‘placeholder’ on the X axes is required to permit visualisation. Horizontal and vertical dashed lines represent a BMR of 50% and BMD_50_, respectively. Individual model fits to each dose-response dataset are shown in Figure S3.

Figure S3—BMD model fits to dose-response data from the DNA-damage ToxTracker endpoints for 17 compounds. Combined datasets were analysed using ‘compound’ as covariate. In each instance, the data were adequately described using a single exponential curve with constant parameters for max-response (*c*) and log-steepness (*d*). Note that the control group (*i.e.,* dose zero) is situated at minus infinity on a log10-scale and a ‘placeholder’ on the X axes is required to permit visualisation. Horizontal and vertical dashed lines represent a BMR of 50% and BMD_50_, respectively.

Figure S4—BMD analyses using exponential or Hill model families for 11 compounds using dose-response data from the *in vivo* micronucleus assay. Combined datasets were analysed using ‘compound’ as covariate. In each instance, the data were adequately described using a single exponential curve with constant parameters for max-response (*c*) and log-steepness (*d*). Note that the control group (*i.e.,* dose zero) is situated at minus infinity on a log10-scale and a ‘placeholder’ on the X axes is required to permit visualisation. Horizontal and vertical dashed lines represent a BMR of 50% and BMD_50_, respectively. Individual model fits to each dose-response dataset are shown in Figure S5.

Figure S5—BMD model fits to dose-response data from the *in vivo* micronucleus assay across 11 compounds. Combined datasets were analysed using ‘compound’ as covariate. In each instance, the data were adequately described using a single exponential curve with constant parameters for max-response (*c*) and log-steepness (*d*). Note that the control group (*i.e.,* dose zero) is situated at minus infinity on a log10-scale and a ‘placeholder’ on the X axes is required to permit visualisation. Horizontal and vertical dashed lines represent a BMR of 50% and BMD_50_, respectively.

Figure S6—BMD-derived potency rankings for 17 compounds obtained using independent fitting of each compound’s dose-response data using the exponential model family. The upper panels show the two-sided, 90% confidence intervals (CIs) for the BMD_50_ defined for each compound using dose-response data from the (A) BSCL2-GFP, (B) RTKN-GFP or (C) BTG2-GFP ToxTracker reporters. The suffix ‘.S9’ indicates compounds tested using supplemental metabolic activation. Dashed CIs represent BMDs where the upper confidence limit of the BMD (*i.e.,* the BMDU) could not be determined from the available dose-response data. Missing CIs reflect BMDs for which the dose-response data did not permit estimation of either the BMDL or the BMDU (i.e. infinite lower and upper CIs). The compound potency ranking (assigned by BMD midpoint) is shown for each reporter down the right-hand side of each output. In reading the potency ranking, overlapping confidence intervals across compounds reflect potencies that cannot be resolved due to uncertainties in the available dose-response data. Even when model fits are carried out entirely independently compound-by-compound, the resultant potency rankings are similar across the three ToxTracker endpoints that report on DNA damage.

Figure S7—Comparison of BMD-derived potency rankings for 17 compounds using dose-response data from ToxTracker cell lines with reporters for either DNA damage (A-C) or protein stress (D). The panels show the two-sided 90% confidence intervals (CIs) for the BMD_50_ defined for each compound using dose-response data from the (A) BSCL2-GFP, (B) RTKN-GFP, (C) BTG2-GFP, or (D) DDIT3-GFP ToxTracker reporters. The suffix ‘.S9’ indicates compounds tested using supplemental metabolic activation. For each compound, the upper and lower intervals reflect the BMD results obtained using the exponential or the Hill model families, respectively. Dashed CIs represent BMDs where the upper confidence limit of the BMD (*i.e.,* the BMDU) could not be determined from the available dose-response data. Missing CIs reflect BMDs for which the dose-response data did not permit estimation of either the BMDL or the BMDU (*i.e.,* infinite lower and upper CIs). In reading the potency ranking, overlapping confidence intervals between compounds reflect potencies that cannot be resolved due to uncertainties in the available dose-response data. Whereas similar potency rankings were established from (A-C) all three DNA damage reporters, comparison against the ranking obtained from the (D) protein stress endpoint shows distinct differences.

geab020_suppl_Supplementary_Figure_S1

geab020_suppl_Supplementary_Figure_S2

geab020_suppl_Supplementary_Figure_S3

geab020_suppl_Supplementary_Figure_S4

geab020_suppl_Supplementary_Figure_S5

geab020_suppl_Supplementary_Figure_S6

geab020_suppl_Supplementary_Figure_S7
