# Supplementary figures and images for: Empirical comparison of genotoxic potency estimations: the in vitro DNA-damage ToxTracker endpoints versus the in vivo micronucleus assay
Source: Mutagenesis. 2021 Jun 10;36(4):311–20. doi: 10.1093/mutage/geab020 (PMC8391785; doi:10.1093/mutage/geab020)

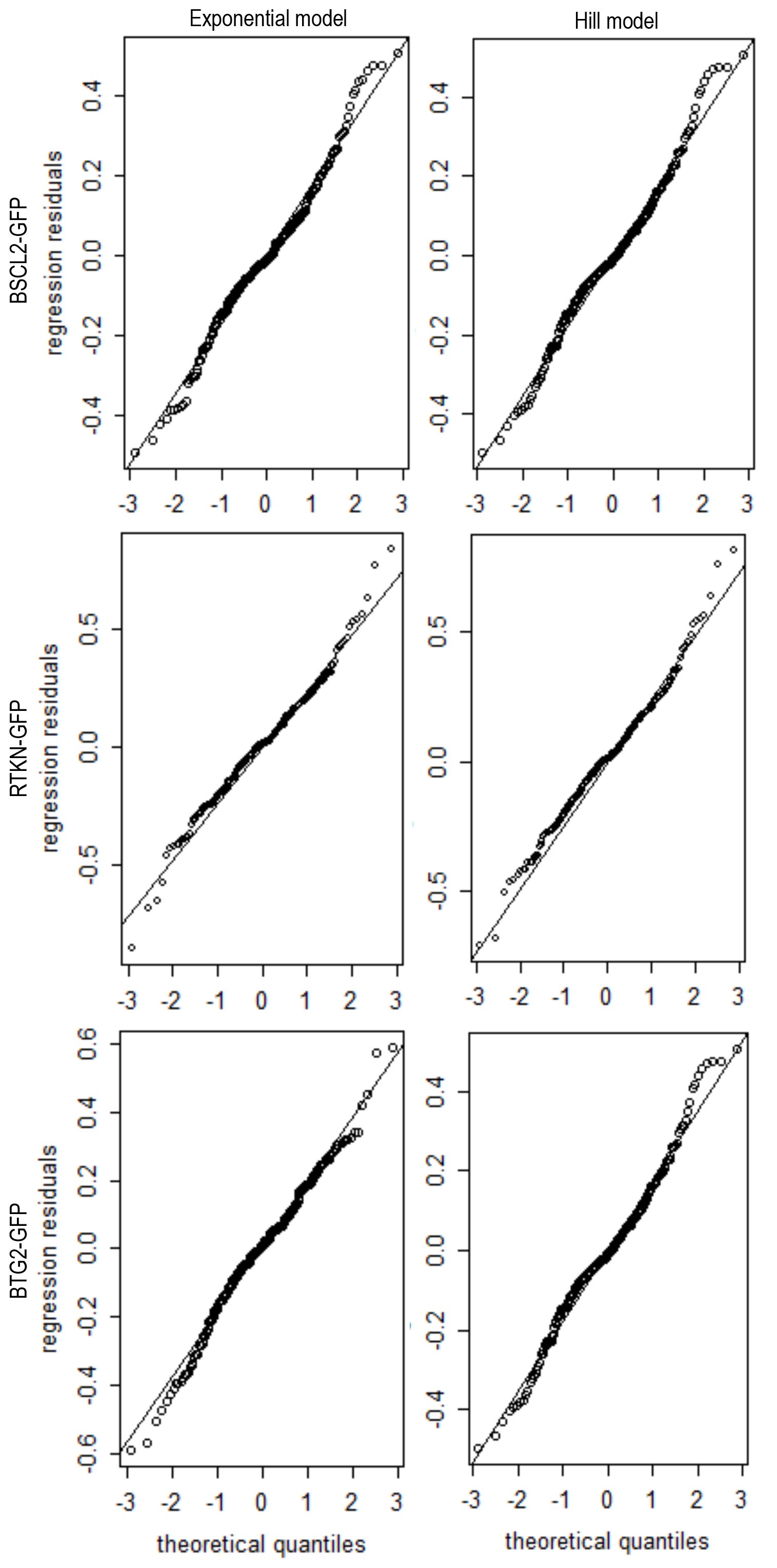

Supplement: geab020_suppl_Supplementary_Figure_S1 [file geab020_suppl_supplementary_figure_s1.jpeg]

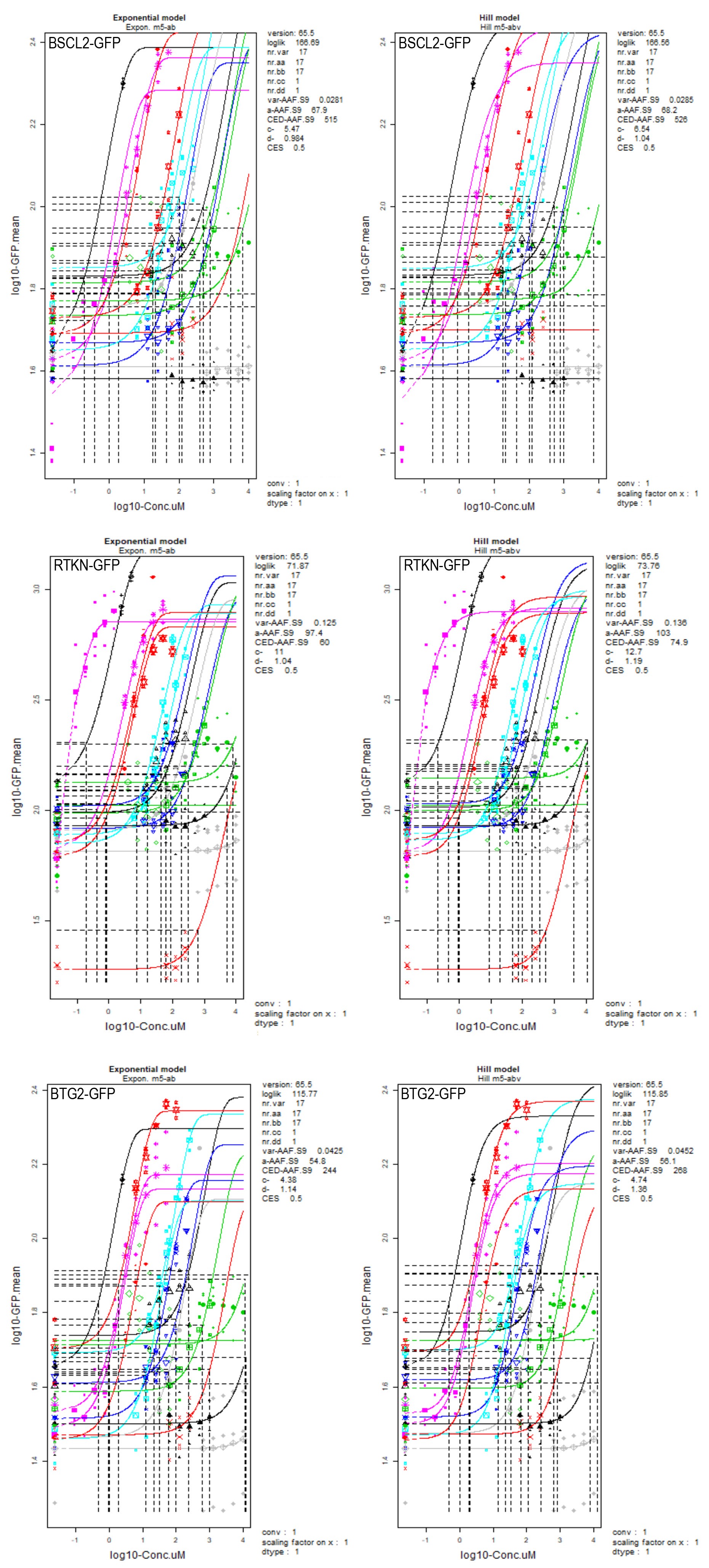

Supplement: geab020_suppl_Supplementary_Figure_S2 [file geab020_suppl_supplementary_figure_s2.jpeg]

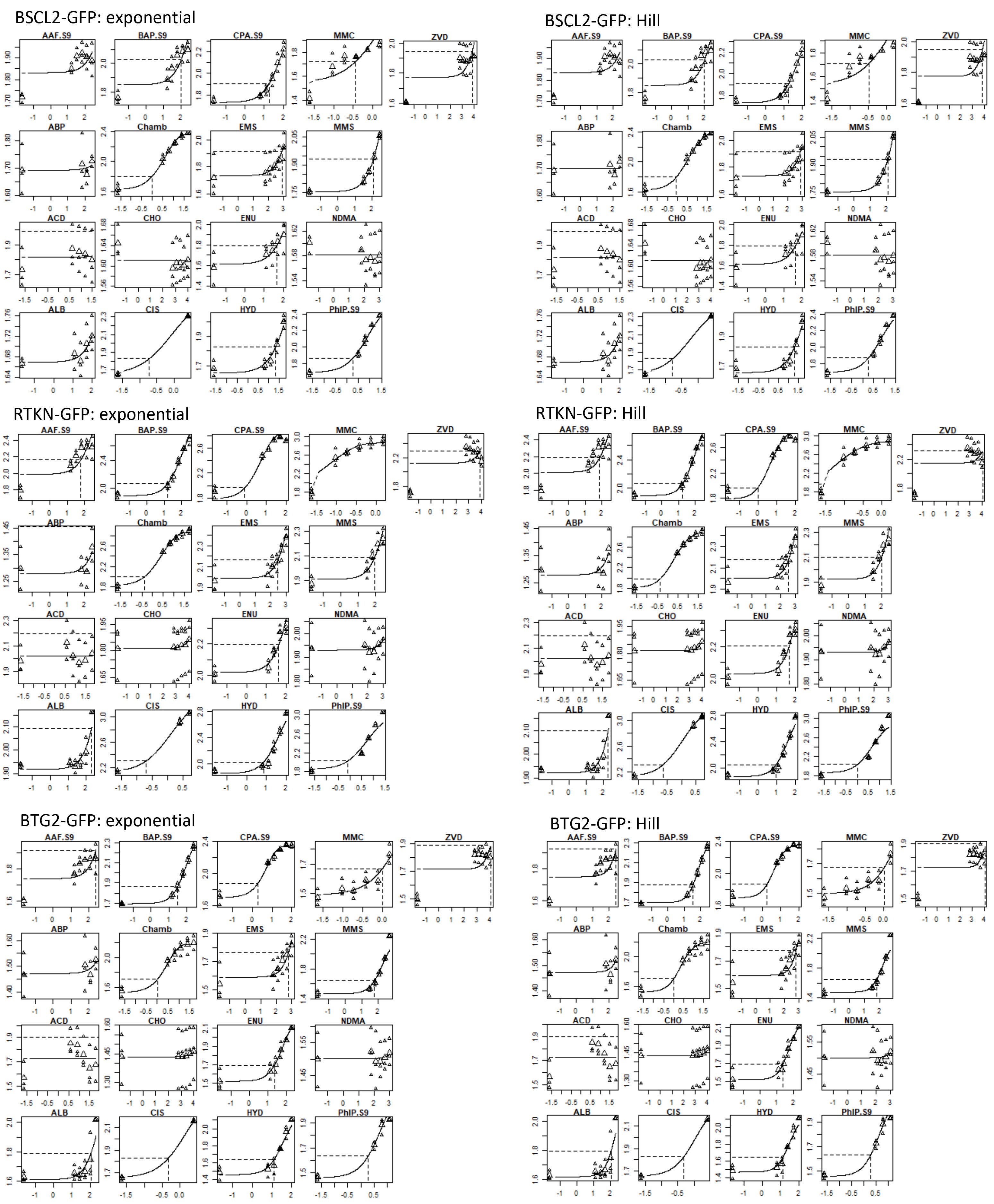

Supplement: geab020_suppl_Supplementary_Figure_S3 [file geab020_suppl_supplementary_figure_s3.jpeg]

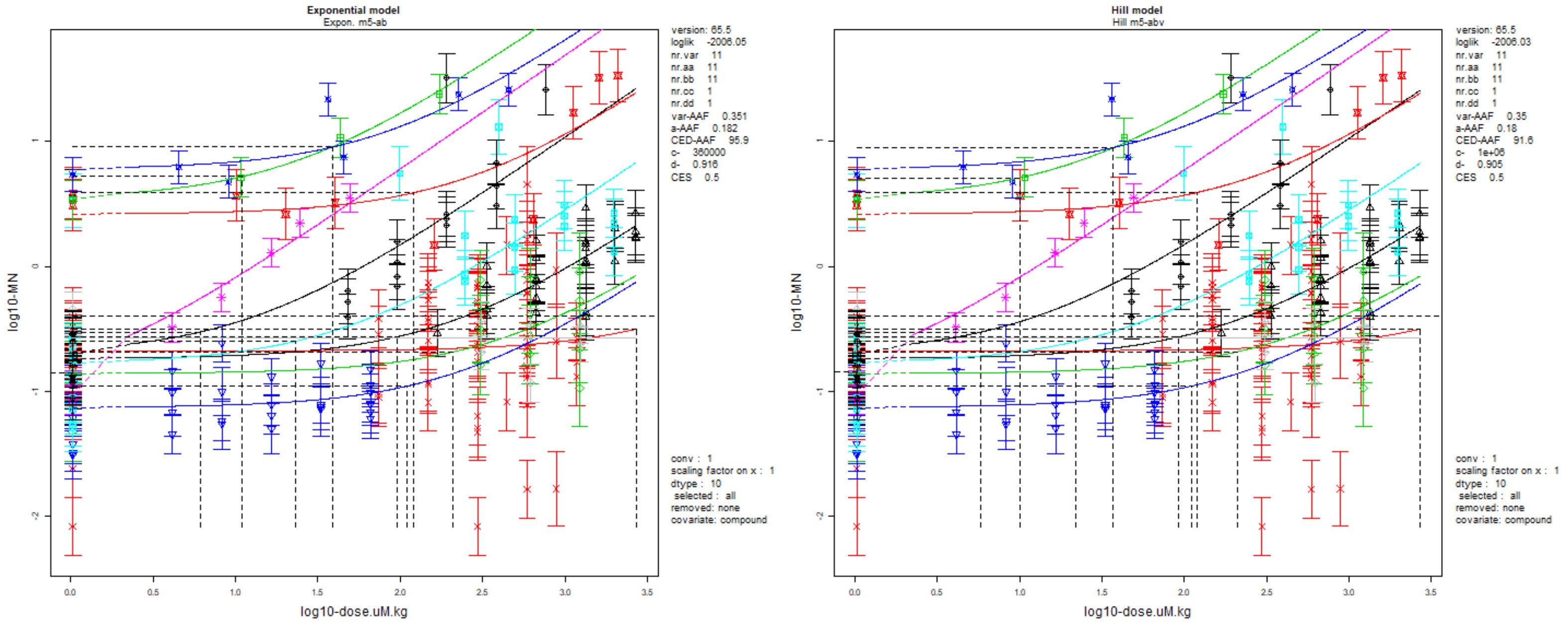

Supplement: geab020_suppl_Supplementary_Figure_S4 [file geab020_suppl_supplementary_figure_s4.jpeg]

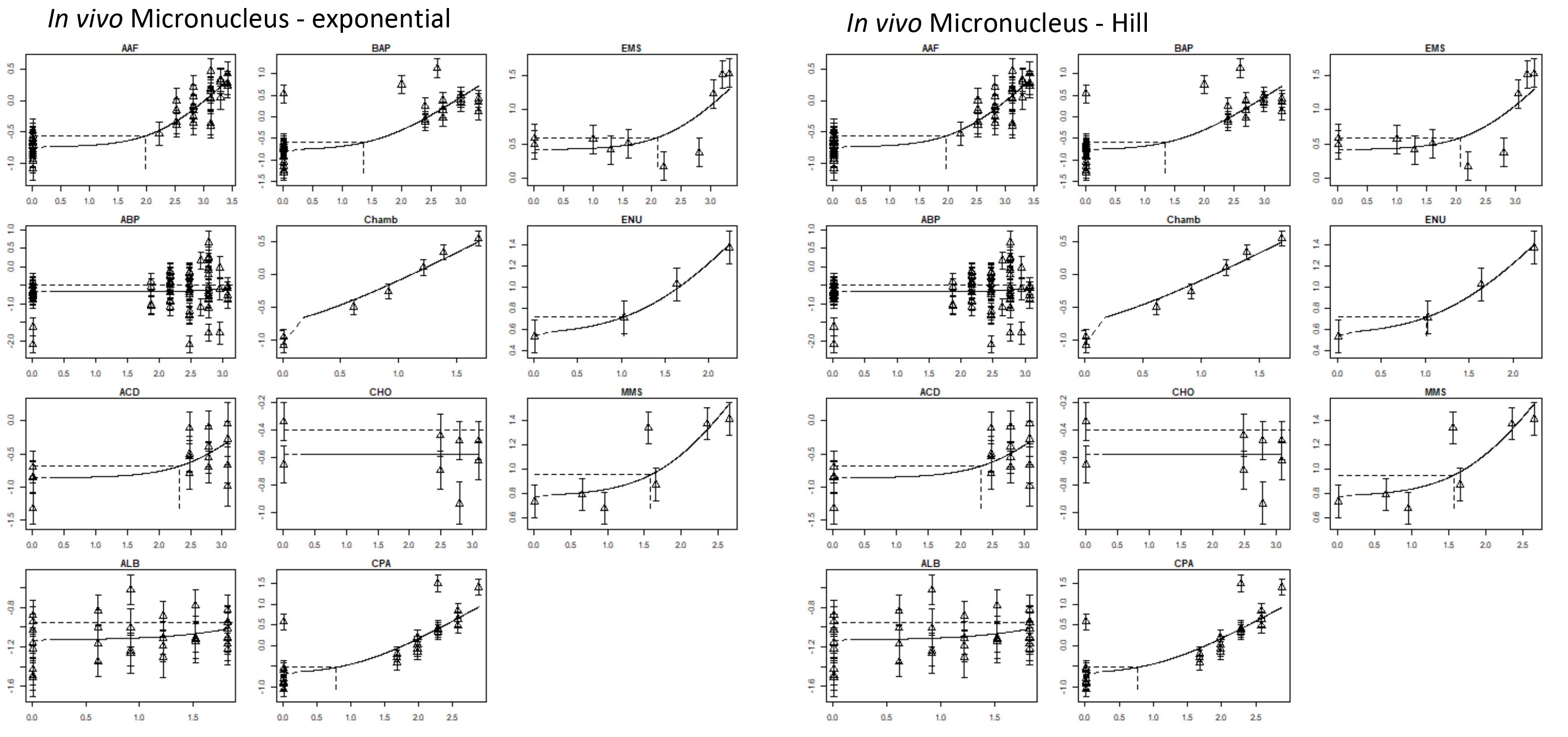

Supplement: geab020_suppl_Supplementary_Figure_S5 [file geab020_suppl_supplementary_figure_s5.jpeg]

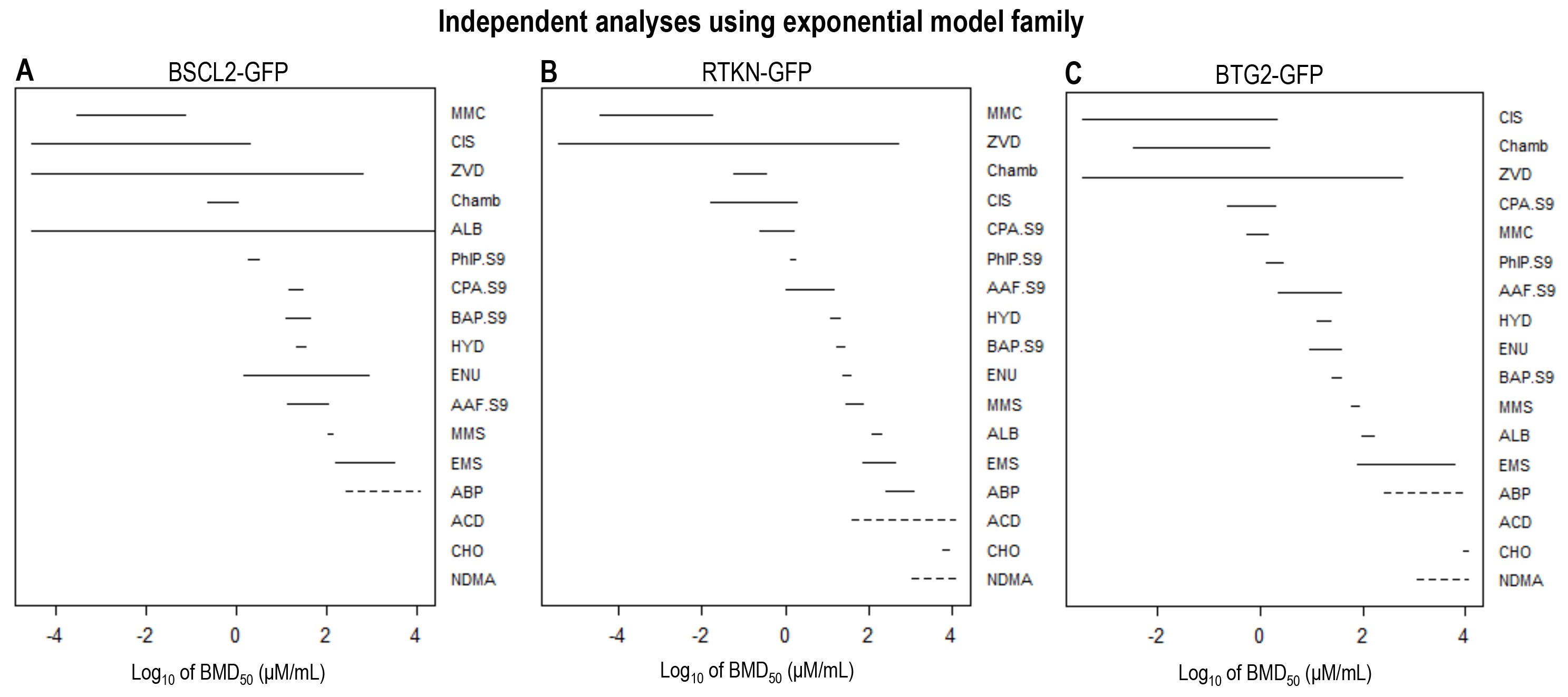

Supplement: geab020_suppl_Supplementary_Figure_S6 [file geab020_suppl_supplementary_figure_s6.jpeg]

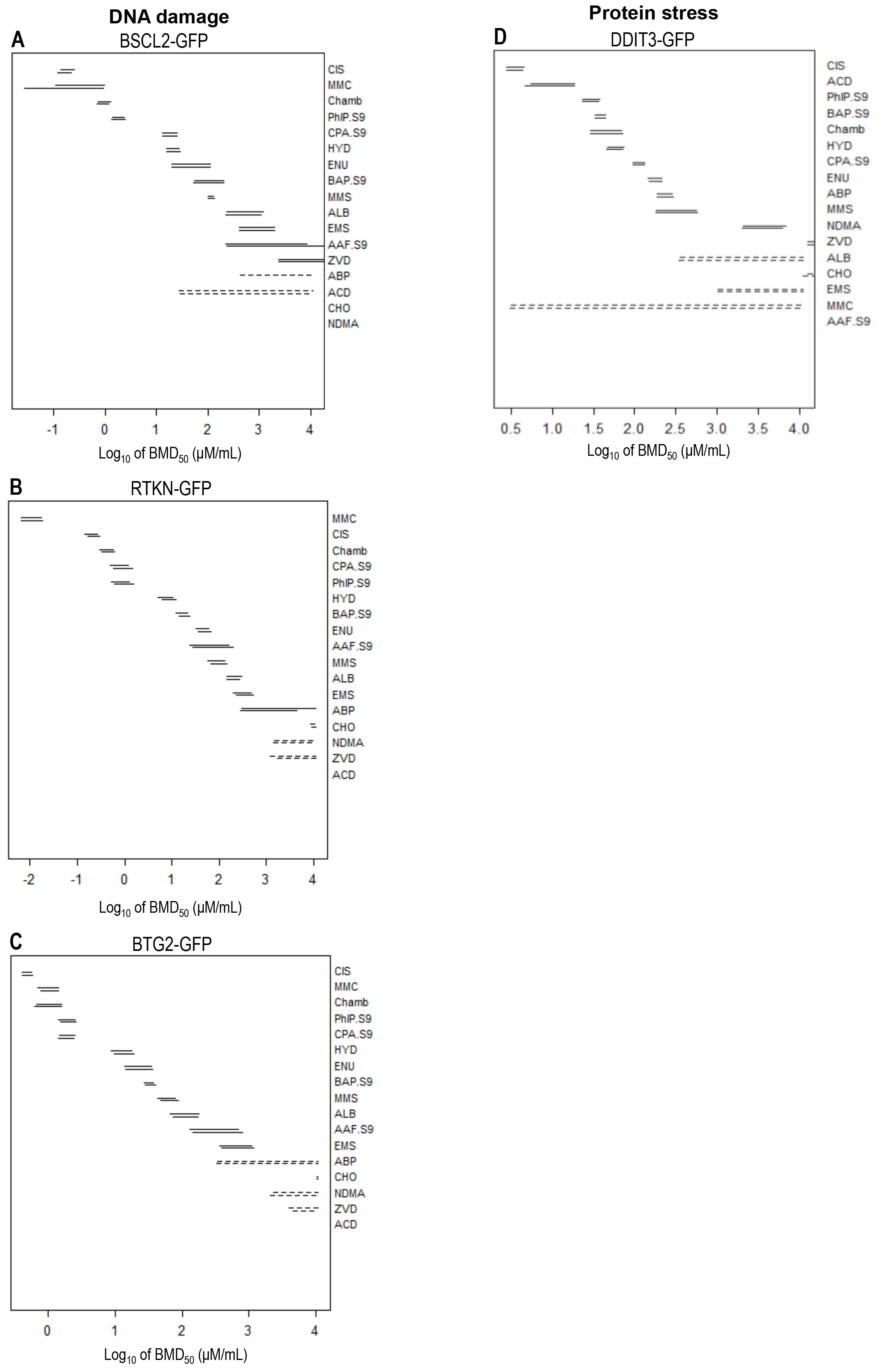

Supplement: geab020_suppl_Supplementary_Figure_S7 [file geab020_suppl_supplementary_figure_s7.jpeg]
